# Advances Toward Engineering Functionally Mature Human Pluripotent Stem Cell-Derived β Cells

**DOI:** 10.3389/fbioe.2020.00786

**Published:** 2020-07-09

**Authors:** Leonardo Velazco-Cruz, Madeleine M. Goedegebuure, Jeffrey R. Millman

**Affiliations:** ^1^Division of Endocrinology, Metabolism and Lipid Research, Washington University School of Medicine, St. Louis, MO, United States; ^2^Department of Biomedical Engineering, Washington University in St. Louis, St. Louis, MO, United States

**Keywords:** stem cells, diabetes, differentiation, pluripotent, transplantation

## Abstract

Human stem cell-derived β (SC-β) cells have the potential to revolutionize diabetes treatment through disease modeling, drug screening, and cellular therapy. SC-β cells are likely to represent an early clinical translation of differentiated human pluripotent stem cells (hPSC). In 2014, two groups generated the first *in vitro*-differentiated glucose-responsive SC-β cells, but their functional maturation at the time was low. This review will discuss recent advances in the engineering of SC-β cells to understand and improve SC-β cell differentiation and functional maturation, particularly new differentiation strategies achieving dynamic glucose-responsive insulin secretion with rapid correction to normoglycemia when transplanted into diabetic mice.

## Introduction

Diabetes mellitus can be characterized as a disease of the β cell, which results in improper insulin secretion and failure to maintain normal glycemia. Type 1 diabetes (T1D) is the result of the dysregulated autoimmune destruction of β cells (Gillespie, 2006). Type 2 diabetes (T2D) is characterized by β cell malfunction and depletion due to high stress caused by chronic hyperglycemia. Currently T1D and many T2D patients are reliant on exogenous insulin treatment. Exogenous insulin treatment requires constant monitoring and injections throughout the day, reducing quality of life and failing to accurately maintain normal glycemia leading to secondary complications (Caro et al., 2002; Powers and D’Alessio, 2011). Transplantation of whole pancreas or purified islets of Langerhans have been shown to result in exogenous insulin independence with accurate glycemic regulation in T1D and T2D patients (Posselt et al., 2010; Gruessner and Gruessner, 2016; Kandaswamy et al., 2018). Widespread application of islet transplantation is limited by replacement tissue availability and the need for immunosuppression (Millman and Pagliuca, 2017).

Human SC-β cells are a promising alternative cell source for diabetes cell replacement therapy as well as disease modeling and drug screening (Millman and Pagliuca, 2017). *In vitro* differentiated SC-β cells were first produced in 2014 (Pagliuca et al., 2014; Rezania et al., 2014). These early SC-β cells presented β cell hallmarks, such as insulin secretion in response to glucose, expression of β cell transcription factors, and *in vivo* function in weeks after transplantation in mice. However, critical differences remained between SC-β cells and primary adult β cells, including inferior insulin secretion per cell, dysregulated glucose-stimulated insulin secretion (GSIS) dynamics, and lower expression of key β cell transcription factors. Recent studies, discussed here, have significantly advanced the functional maturity of SC-β cells resulting in functionally enhanced SC-β cells. These enhanced SC-β cells have improved function, with some achieving dynamic insulin secretion marked by the presence of first and second phase insulin secretion. Despite the enhancement of these SC-β cells, they fail to match the glucose responsiveness and transcriptomic profile of primary cadaveric islets (Baron et al., 2016; Nair et al., 2019; Velazco-Cruz et al., 2019; Hogrebe et al., 2020; Mahaddalkar et al., 2020). In this review we describe the advancements made for achieving enhanced SC-β cells and the path toward differentiating fully functional SC-β cells resembling cadaveric islets in terms of their function and transcriptomic profile.

## The Path Toward SC-β Cells

The path toward differentiating SC-β cells has proved challenging, having already spanned over two decades. Progress has occurred in waives as hard-fought milestones are achieved. Early pioneering work established methodologies for differentiating hPSCs toward definitive endoderm (D’Amour et al., 2005), the first developmental stage in the path toward making β cells. Further sequential treatments of growth factors and small molecules continued to mimic pancreatic organogenesis guiding hPSCs through stages resembling definitive endoderm, gut-tube endoderm, pancreatic endoderm, and ultimately hormone expressing endoderm. The resulting insulin producing cells were polyhormonal, failed to maintain PDX1 and NKX6.1 expression, and were not glucose responsive (D’Amour et al., 2006). However, transplantation of hPSC-derived pancreatic progenitors into immunocompromised mice allowed for their differentiation into monohormonal glucose-stimulated insulin-secreting cells after several months *in vivo* (Kroon et al., 2008; Rezania et al., 2012). Since then, other groups have demonstrated that PDX1 and NKX6.1 expressing pancreatic progenitors have the potential of differentiating toward β cells (Rezania et al., 2013; Schaffer et al., 2013; Nostro et al., 2015; Millman et al., 2016).

In 2014, two protocols were published for the efficient generation of glucose-responsive monohormonal *in vitro*-differentiated SC-β cells (Pagliuca et al., 2014; Rezania et al., 2014). These protocols generated pancreatic progenitors with high PDX1, NKX6.1 and low NGN3 expression. Low NGN3 expression through Vitamin C treatment distinguishes these pancreatic progenitors from earlier protocols (Kroon et al., 2008; Rezania et al., 2012). These pancreatic progenitors where then differentiated to hormone expressing endocrine cells through transient NGN3 upregulation by treatment with TGFβR1 inhibitor ALK5i Type II (ALK5i) and thyroid hormone (T3). Air-liquid interface culture was observed to increase NGN3 expression relative to planar culture (Rezania et al., 2014) while the other protocol was completed in suspension culture (Pagliuca et al., 2014). The continued treatment of endocrine CHGA+ cells with ALK5i, T3, and γ-secretase (XXI or XX) drives the endocrine population toward monohormonal INS+ cells. The final stage of these protocols cultured the cells with ALK5i and T3 resulting in glucose responsive SC-β cells making up ∼40% of the population. In addition to ALK5i and T3, Rezania includes compounds R428, an AXL inhibitor, with N-acetyl cysteine, claiming them to drive expression of β cell maturation gene MAFA. The Rezania et al. air-liquid interface culture format can be more easily replicated by laboratories with standard culture experience and equipment, however it is less scalable than the suspension culture format described in Pagliuca et al. which requires more specialized equipment and knowledge of 3D cell culture. These original SC-β cells represented a monumental accomplishment being scalable, glucose responsive, transcriptionally similar to primary islets, and capable of regulating mouse blood glucose in weeks post transplantation. Despite these accomplishments the resulting SC-β cells were functionally immature, secreting low levels of insulin, no dynamic insulin secretion, immature calcium response, and transcriptional differences remained relative to cadaveric islets with measurable differences in MAFA, UCN3, and GCK gene expression (Pagliuca et al., 2014; Rezania et al., 2014).

## Advancing SC-β Cells

Early SC-β cells are functionally immature lacking dynamic insulin secretion and observable functional maturation occurring after transplantation *in vivo*, marked by an increase in secreted insulin with time post transplantation (Pagliuca et al., 2014; Rezania et al., 2014; Russ et al., 2015). More functional SC-β cells are needed to improve cell replacement outcomes and facilitate more robust disease modeling studies. Recent publications have demonstrated improved differentiation efficiency, higher glucose stimulated insulin secretion, first and second phase insulin secretion, response to multiple secretagogues, and fast *in vivo* glucose regulation upon transplantation (Table 1; Nair et al., 2019; Velazco-Cruz et al., 2019; Veres et al., 2019; Hogrebe et al., 2020; Mahaddalkar et al., 2020).

**TABLE 1:** Characteristics of Enhanced SC-β cell protocols.

**Article**	**Format**	**Reaggregation**	**Sorting**	**Endocrine**	**SC-β Cell**	**1st Phase stimulation**	**2nd Phase stimulation**	**Return to baseline insulin secretion at low glucose**	**Insulin content μ IU insulin/1,000 cells**
Velazco-Cruz et al., 2019	S	Yes	n/a	>95%	52%	7.6	2.1	Yes	321
Veres et al., 2019	S	Yes	CD49A + S6	>95%	80%	3.2	1.5	No	N.R.
Nair et al., 2019	S	Yes	INS:GFP S6	>99%	80%	3.5	1	n/a	N.R.
Hogrebe et al., 2020	P	Yes	n/a	>95%	31%	9.4	1.9	Yes	150
Mahaddalkar et al., 2020	S	Yes	CD177 + S1	N.R.	62%	5	1.15	n/a	317

*Quantitative values estimated from graphs when not reported in text. N.R., not reported; S, suspension; P, planar; S#, stage #.*

Velazco-Cruz et al. was first to report robust dynamic insulin secretion of SC-β cells (Velazco-Cruz et al., 2019) with both first and second phase kinetics using a suspension differentiation protocol with temporal TGFβ modulation, cellular cluster size control, serum free media, endocrine enrichment without cell selection, and a simplified final stage media lacking notable prior factors (T3, N-acetyl cysteine, Trolox, and R428). The authors show that treatment with TGFβR1 inhibitor, ALK5 inhibitor type II (ALK5i), is necessary for specification of the β cell fate. However, upon β cell specification permittance of TGFβ signaling is critical for SC-β cell functional maturation. However, ALK5i is widely used in the final stage of many β cell differentiation protocols (Pagliuca et al., 2014). The authors show that by re-sizing cellular clusters during the last stage of the differentiation, a process which involves partial dissociation of clusters as previously reported (Song and Millman, 2017), enhances static GSIS and nearly doubles the C-Peptide+ NKX6.1+ co-expressing SC-β cell population. In dynamic secretion assays, robust dynamic function with a clear first phase, stable second phase, and a return to basal levels after high glucose treatment ends was shown. However, a weakness of the study was that insulin secretion per cell and the degree insulin secretion was increased in response to high glucose varied across different hPSC lines. Transplantation of these cells improved glucose tolerance in mice.

Veres et al. employed some of the same changes as Velazco-Cruz et al., including cellular cluster size control, serum free media, endocrine enrichment without cell selection, and a final stage media lacking T3, N-acetyl cysteine, Trolox, and R428 (Veres et al., 2019). Additionally, Veres et al. employs β cell enrichment using cell sorting. Through a similar cellular reaggregation method, Veres et al. sees strong endocrine enrichment and an increase in the frequency of the C-Peptide+ /NKX6.1+ SC-β cell population along with improved static GSIS. The authors further show that enrichment of the β cell population through CD49a+ cell sorting improved static GSIS relative to unsorted and reaggregated SC-β cells. CD49a+ sorted cells demonstrated first and second phase insulin secretion, however a return to basal levels did not occur when high glucose was removed. The authors did not show if reaggregated cells could undergo dynamic GSIS. The authors identify enterochromaffin-like cells in their SC-β cell preps and CD49+ sorting removes these cell types. Enterochromaffin cells, marked by TPH1 expression, secrete serotonin in the gut and are absent from the human pancreas. This study indicates β cells and enterochromaffin cells share a similar developmental path resulting in their presence in β cell differentiation protocols. It is unclear if the functional benefits observed by CD49+ sorting are due to the removal of off target cell types, such as enterochromaffin-like cells, or other mechanisms. Transplantation into mice was not investigated in this study.

In a separate study, Nair et al. achieved first phase insulin secretion using an insulin-driven GFP tag cell sorting approach and a reaggregation process (Nair et al., 2019). The authors achieved a high β cell population with 80% C-Peptide/NKX6.1 co-expression. In functional studies the authors compare reaggregated GFP enriched cells to un-reaggregated unsorted β cell clusters, with limited functional studies using reaggregated and unsorted clusters as a control. The authors see no first phase response in the immature clusters or the reaggregated and unsorted clusters while there GFP sorted reaggregated clusters have a first phase stimulation index of ∼3 and no second phase response. However, the study design did not allow for distinguishing functional improvements related to endocrine enrichment by reaggregation or β cell enrichment by sorting, like done with CD49a enrichment (Veres et al., 2019). This study was performed using only one insulin-driven GFP reporter cell line, making it unclear how well this approach would apply to other cell lines and whether the low levels of insulin secretion are due to the genetic engineering or genetic background of this cell line relative to other protocols. It is important to note that this differentiation protocol retains ALK5i and T3 during the final stage of differentiation, while other enhanced SC-β cell protocols have removed them (Rosado-Olivieri et al., 2019; Velazco-Cruz et al., 2019; Veres et al., 2019; Hogrebe et al., 2020; Maxwell et al., 2020). Therefore, it is possible that removal of these compound will allow for more robust dynamic secretion including the missing second phase observed by this protocol. Transplantation of these cells improved glucose tolerance in mice.

Hogrebe et al. (2020) used a different differentiation strategy for generating SC-β cells, demonstrating that regulation of actin cytoskeleton polymerization controls differentiation to endocrine and other endodermal lineages. This insight led to development of a planar β cell differentiation protocol. Other protocols use suspension (Pagliuca et al., 2014; Russ et al., 2015; Rosado-Olivieri et al., 2019; Velazco-Cruz et al., 2019; Veres et al., 2019) or pseudo-suspension air-liquid interface (Rezania et al., 2014) culture methodologies for endocrine specification, increasing the technical expertise required for SC-β cell differentiations. Using a novel planar differentiation protocol with actin depolymerizer latrunculin A driving endocrine specification, through neurogenin three upregulation, the authors generate SC-β cells which undergo dynamic GSIS. When these SC-β cells are transplanted into mice, they rapidly reversed severe preexisting diabetes at a rate resembling that achieved by cadaveric islets.

In a controlled and parallel fashion, Hogrebe et al. compared his planar differentiated β cells to suspension differentiated β cells using the Velazco-Cruz et al. protocol. Using the HUES8 cell line, for which the two protocols were optimized, the suspension protocols had higher percent yields of CP+ NKX6.1+ SC-β cells. Functionally, planar and suspension derived SC-β cells were similar when assayed by static and dynamic GSIS as well as insulin content. When assayed by real-time PCR, the planar and suspension derived SC-β cells were similar. An equal number of planar and suspension derived SC-β cells were transplanted into diabetic mice. Diabetes reversal with planar differentiated β cells occurred in two weeks, while the suspension protocol took 5 weeks. This discrepancy in diabetes cure speed is interesting, as *in vitro* functional and transcriptomic assays did not show evident differences between the two protocols and based on reported differentiation efficiencies the suspension protocol generates a higher proportion of SC-β cells. Single-cell RNA sequencing, comparing transcriptomes of planar and suspension derived SC-β cells could reveal further insights into the source of this discrepancy. Importantly, the Hogrebe et al. planar protocol was able to successfully differentiate SC-β cells from multiple pluripotent stem cell lines, with some cell lines matching cadaveric islets in function when assayed with dynamic perfusion assays. While the HUES8 suspension and planar derived SC-β cells were functionally similar, the planar protocol yielded higher functioning cells when applied to different cell lines. The robustness of the planar Hogrebe et al. differentiation protocol facilitates studies using more than one cell line (Maxwell et al., 2020; Velazco-Cruz et al., 2020).

Using a sorting approach for CD177/NB1 glycoprotein, Mahaddalkar et al. isolated anterior definitive endoderm cells with increased pancreatic and β cell potential (Mahaddalkar et al., 2020). The authors characterize CD177+ cells to have increase PDX1 and NKX6.1 pancreatic progenitor potential when compared to unsorted and CD275+ definitive endoderm populations. Additionally, this work shows canonical WNT inhibition by IWP2 treatment to increase pancreatic progenitor differentiation efficiency, a finding supported by previous publications (Loh et al., 2014; Davenport et al., 2016; Zhu et al., 2016). Differentiation of CD177+ cells toward β cells resulted in improved differentiation efficiency and function relative to unsorted cells. CD177+ cells presented a first phase insulin response with no second phase, while unsorted cells did not present a first or second phase insulin secretion (Mahaddalkar et al., 2020). The cells were not transplanted into mice.

Direct comparison of these studies is difficult, as assays evaluating function are variable, including technical methodologies, normalization strategies, and *in vivo* models differ. Normalizing SC-β cells to cadaveric human islet insulin secretion is imperfect, as cadaveric islet function is highly variable within and between studies (Pagliuca et al., 2014; Nair et al., 2019; Velazco-Cruz et al., 2019; Veres et al., 2019). Standardized static and dynamic GSIS assays, normalized to DNA, can greatly facilitate comparison of differentiation protocols while imposing minimal burden on investigators. Standardization of *in vivo* assays are more challenging, as many mouse and diabetic models exist with variable severity of diabetes. By providing standardized *in vitro* GSIS results, individual researchers can better compare protocols and guide the differentiation protocols employed in their studies.

## Forging a Path Forward

Despite advances, current SC-β cells lack the functional maturity of cadaveric islets. In the continuing quest to functionally mature SC-β cells, teams are employing novel technologies and approaches, such as single-cell sequencing, genetic engineering, cell sorting, and drug screening, to identify factors which contribute to β cell differentiation and function. Recent publications have compared gene expression between adult and fetal or juvenile islets, with many adult genes having potential roles in the functional maturation of SC-β cells. ERRγ has been characterized to be enriched in adult vs neonatal mouse β cells and mice deficient of β cell ERRγ fail to properly regulate their blood glucose (Yoshihara et al., 2016). Yoshira et al. differentiates hPSCs toward an immature β-like state in which many β cells genes are expressed but are incapable of undergoing *in vitro* GSIS. The authors overexpress ERRγ in these β-like cells and observed improvements to mitochondrial respiration and the β-like cells gain the ability to undergo *in vitro* GSIS. Upregulation of ERRγ can potentially be used to further mature SC-β cells. However, since its effects were only observed in immature insulin-expressing endocrine cells incapable of undergoing *in vitro* GSIS with immature mitochondrial respiration, it may not translate to more advanced protocols which are more metabolically mature (Nair et al., 2019). In a separate study, Arda et al. shows islet function increases in adult vs juvenile human islets identifying several genes associated with age in β cells including ONECUT2, MAFA, TSHZ3, SIX2, and SIX3 (Arda et al., 2016). It remains to be investigated whether upregulation of these genes in SC-β cells can drive their functional maturation.

Inhibition of YAP signaling has been shown to drive endocrine induction through neurogenin three upregulation and when incorporated into differentiation protocols during endocrine induction, using verteporfin, β differentiation efficiency and function is enhanced (Rosado-Olivieri et al., 2019). Dynamic function is not assayed in this work. This work is supported by a previous study showing pancreatic progenitor endocrinogenesis is stimulated by YAP inhibition (Mamidi et al., 2018). Incorporation of YAP inhibitors during endocrine specification and YAP agonist during β cell maturation may drive improvements to β cell generation.

Recent work (Ameri et al., 2017; Cogger et al., 2017; Nair et al., 2019; Veres et al., 2019; Mahaddalkar et al., 2020) has shown that enrichment of certain cell populations can ultimately improve β cell differentiation efficiency and function. Transcriptomic (Ameri et al., 2017) and proteomic (Cogger et al., 2017) approaches have revealed glycoprotein two as a cell surface marker beneficial for sorting PDX1+/NKX6.1+ pancreatic progenitors improving β cell differentiation efficiency. Although functional improvements were not seen, this strategy may increase the proportion of β cells generated using enhanced differentiation protocols. Enrichment of the β cell population through sorting may enhance the functional maturation of SC-β cells (Nair et al., 2019; Veres et al., 2019). Whether this improvement is through cell-cell contact, paracrine signaling, or the removal of inhibitory cell types, such as enterochromaffin cells (Veres et al., 2019), remains to be determined with more robust studies necessary. Cell enrichment using cell sorting limits large scale production of SC-β cells. However, the scale of production necessary for SC-β cells may be compatible with magnetic-activated cell sorting approaches, particularly as they have proven beneficial. Additionally, identification of markers such as glycoprotein two can guide the search for small molecules to increase the population of cells expressing the desired markers. High throughput screening for small molecules affecting differentiation has been successful, with the identification of Rho-kinase inhibitor H1152, capable of increasing MAFA and UCN3 expression (Ghazizadeh et al., 2017).

Single-cell sequencing technologies are rapidly advancing our understanding of β cell fate, differentiation, and functional maturation. Several studies have increasingly elucidated the β cell transcriptome (Baron et al., 2016; Segerstolpe et al., 2016; Wang et al., 2016; Xin et al., 2016; Veres et al., 2019), revealing novel β cell enriched genes which may be used as markers for driving β cell functional maturation. Recently, single-cell patch-clamp sequencing was used linking β cell gene expression to functional phenotypes revealing sets of genes correlating with β cell function (Camunas-Soler et al., 2020). Veres et al. (2019) performed single-cell sequencing at multiple stages during SC-β cell differentiations revealing transcriptomic profiles of each stage and genes whose expression is correlated to the acquisition of function by SC-β cells. Epigenome analysis of SC-β cell differentiation reveal LMX1B as a regulator of endocrine specification and circadian rhythms as a component toward SC-β cell functional maturation (Alvarez-Dominguez et al., 2020). These studies give researchers a more accurate β cell transcriptomic model to guide differentiation protocol development. To further advance SC-β cell technology, researchers must continue efforts to build our understanding of β cell development which guides development of β cell differentiation protocols.

## Discussion

SC-β cells are a promising cell source for diabetes cell therapy, disease modeling, and understanding human development. The use of small molecules and growth factors to drive stem cell differentiation toward β cells, mimicking *in vivo* development, has proven a reliable strategy amenable to scale-up and genetic perturbations. Since the first fully *in vitro* differentiation protocols (Pagliuca et al., 2014; Rezania et al., 2014) capable of generating glucose responsive β cells, the field has significantly advanced. Through the optimization of differentiation protocols, including the removal of ALK5i during the final stage of differentiation and reaggregation driven endocrine enrichment, enhanced SC-β cells have greater glucose responsiveness undergoing dynamic GSIS. Currently, SC-β cells are still less functional than cadaveric islets secreting lower amounts of insulin and a stable but lower in magnitude second phase insulin secretion (Nair et al., 2019; Velazco-Cruz et al., 2019; Veres et al., 2019). Transcriptionally, SC-β cells resemble cadaveric islets more so than fetal islets, however critical differences remain, such as reduced expression of maturation factors MAFA and SIX3 (Veres et al., 2019). Using recently published technologies and approaches our understanding of β cell development is improving, guiding the development of novel protocols capable of generating SC-β cells with function matching that of primary islets. Generation of fully functionally mature SC-β cells may be possible in the next few years and will drive diabetes cell therapies forward while providing a robust model for development and disease modeling.

## Author Contributions

LV-C and JM conceived the manuscript. All authors contributed to the article and approved the submitted version.

## Conflict of Interest

LV-C and JM are inventors in patents and patent applications relating to the differentiation of stem cell-derived β cells. The remaining author declares that the research was conducted in the absence of any commercial or financial relationships that could be construed as a potential conflict of interest.
